# Serum antibodies against genitourinary infectious agents in prostate cancer and benign prostate hyperplasia patients: a case-control study

**DOI:** 10.1186/1471-2407-11-53

**Published:** 2011-02-03

**Authors:** Jan Hrbacek, Michael Urban, Eva Hamsikova, Ruth Tachezy, Vaclav Eis, Marek Brabec, Jiri Heracek

**Affiliations:** 1 Charles University in Prague, 3rd Faculty of Medicine, Department of Urology, Prague, Czech Republic; 2 Institute of Hematology and Blood Transfusion, Prague, Czech Republic; 3 Charles University in Prague, 3rd Faculty of Medicine, Department of Pathology, Prague, Czech Republic; 4 Institute of Computer Science, Academy of Sciences of the Czech Republic, Department of Nonlinear Modelling, Prague, Czech Republic; 5 Androgeos, Prague, Czech Republic

## Abstract

**Background:**

Infection plays a role in the pathogenesis of many human malignancies. Whether prostate cancer (PCa) - an important health issue in the aging male population in the Western world - belongs to these conditions has been a matter of research since the 1970 s. Persistent serum antibodies are a proof of present or past infection. The aim of this study was to compare serum antibodies against genitourinary infectious agents between PCa patients and controls with benign prostate hyperplasia (BPH). We hypothesized that elevated serum antibody levels or higher seroprevalence in PCa patients would suggest an association of genitourinary infection in patient history and elevated PCa risk.

**Methods:**

A total of 434 males who had undergone open prostate surgery in a single institution were included in the study: 329 PCa patients and 105 controls with BPH. The subjects' serum samples were analysed by means of enzyme-linked immunosorbent assay, complement fixation test and indirect immunofluorescence for the presence of antibodies against common genitourinary infectious agents: human papillomavirus (HPV) 6, 11, 16, 18, 31 and 33, herpes simplex virus (HSV) 1 and 2, human cytomegalovirus (CMV), Chlamydia trachomatis, Mycoplasma hominis, Ureaplasma urealyticum, Neisseria gonorrhoeae and Treponema pallidum. Antibody seroprevalence and mean serum antibody levels were compared between cases and controls. Tumour grade and stage were correlated with serological findings.

**Results:**

PCa patients were more likely to harbour antibodies against Ureaplasma urealyticum (odds ratio (OR) 2.06; 95% confidence interval (CI) 1.08-4.28). Men with BPH were more often seropositive for HPV 18 and Chlamydia trachomatis (OR 0.23; 95% CI 0.09-0.61 and OR 0.45; 95% CI 0.21-0.99, respectively) and had higher mean serum CMV antibody levels than PCa patients (p = 0.0004). Among PCa patients, antibodies against HPV 6 were associated with a higher Gleason score (p = 0.0305).

**Conclusions:**

Antibody seropositivity against the analyzed pathogens with the exception of Ureaplasma does not seem to be a risk factor for PCa pathogenesis. The presence or higher levels of serum antibodies against the genitourinary pathogens studied were not consistently associated with PCa. Serostatus was not a predictor of disease stage in the studied population.

## Background

Prostate cancer (PCa) is one of the most important health issues in the aging male population, especially in the industrialized Western world. In the EU in 2006, it accounted for approximately 20% of all noncutaneous cancers [1]. In the United States, 217.730 new cases were estimated to occur in 2010 (28% of all new cancer cases in men except for basal and squamous cell skin carcinomas) and 32.050 men were expected to die from PCa (11% of all cancer-related deaths) [2].

Research on the etiology of human cancer has found evidence for 15-20% of them being caused by an infectious agent. Whether PCa or at least a subgroup of PCa cases are associated with infection has been a matter of debate since the 1970 s. With the widespread use of sophisticated serological assays and molecular biology methods for the detection of infectious agents in tissue, several studies have been published in the past two decades giving a better insight into the matter. Many of them have focused on a single pathogen only [3-9].

We have conducted an epidemiological study of several most common genitourinary pathogens among PCa and benign prostate hyperplasia (BPH) patients. The aim of the study was to elucidate whether the prevalence of antibody seropositivity and/or antibody levels differ between these two groups of patients. We hypothesized that elevated serum antibody levels or higher seroprevalence in PCa patients would suggest an association of genitourinary infection in patient history and elevated PCa risk.

## Methods

### Study population

This is a hospital-based case-control study. The study population consisted of a total of 434 Caucasian males who had been treated with open prostate surgery in the Department of Urology of the 3^rd ^Faculty of Medicine, Charles University in Prague in the period 2004-2010. The institution is a public hospital with a non-selective admission policy based on a defined catchment area. Patients underwent open radical retropubic prostatectomy (RRP) modified by Reiner and Walsh [10,11] for clinically localized PCa or simple transvesical prostatectomy (SP) as described by Fuller and Culp [12,13] for BPH. No transurethral resection of the prostate (TURP) or needle-biopsy specimens were used in the study as both methods only deliver so little tissue that small foci of PCa can be missed. In 5 cases, percutaneous cystolithotripsy was performed along with SP. All patients were free of urinary tract infection symptoms and had a negative urine culture at the time of surgery. Those with untreatable bacteriuria (eg. for bladder stones and/or an indwelling catheter) were treated with antibiotics in the perioperative period.

**Figure 1:**
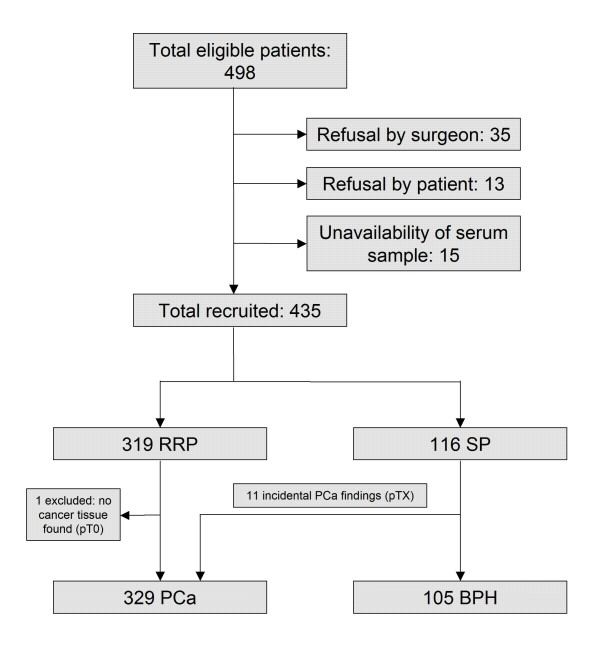
**Patient flow diagram**. A scheme of the patient recruitment process. RRP radical retropubic prostatectomy, SP simple transvesical prostatectomy, PCa prostate cancer, BPH benign prostate hyperplasia.

Study population characteristics are summarized in Table 1. See patient flow diagram (Figure 1) for recruitment and pre-enrolment phase details.

**Table 1:** The study population characteristics

	Patient group	
	
	PCa	BPH
**Total number of patients**	**329 patients**	**105 patients**

Mean age (years)	64 (39-81)	72 (54-87)

<60	76 (23.1%)	7 (6.7%)

60-69	191 (58.1%)	33 (31.4%)

70-79	61 (18.5%)	43 (41.0%)

>79	1 (0.3%)	22 (21.0%)

Mean PSA (ng/mL)	9.0 (0.8-35.4)	7.5 (0.9-39.0)

0-4.0	37 (11.5%)	25 (33.8%)

4.1-10.0	176 (54.8%)	35 (47.3%)

10.1-20.0	94 (29.3%)	9 (12.2%)

>20.0	14 (4.4%)	5 (6.7%)

Overall demographic and clinical characteristics of the studied population. PCa prostate cancer, BPH benign prostate hyperplasia, PSA prostate specific antigen

A written informed consent, approved by the Internal Grant Agency of the Ministry of Health of the Czech Republic and by the Ethical Committee of the 3^rd ^Faculty of Medicine, Charles University in Prague (an IRB-like body which also approved the study), was obtained from all patients.

### Tissue sample handling

Immediately after removal from the anatomical location, the specimens were referred to the Department of Pathology for examination: radical prostatectomy specimens were processed according to the scheme described by Montironi [14]: the external surface of the prostate was painted with ink to indicate surgical margins, seminal vesicles were amputated and embedded. Then the apical and bladder neck margins were removed and sectioned parallel to the urethra and submitted for the examination of margins. The rest of the prostate was sliced at 5 mm intervals perpendicularly to the urethra and examined. Tissue specimens obtained from transvesical prostatectomy were sectioned in 5 mm slices and representative sections from each lobe (a minimum of three sections from each lobe, or one section per 10 g of tissue) were examined [15]. Tissue specimens were routinely fixed with 4% buffered formalin and embedded in paraffin. Microscopic slides were stained with hematoxylin and eosin and evaluated by optical microscopy. A single pathologist experienced in urogenital pathology performed microscopic evaluation of the slides. The morphological parameters were recorded as follows: histological type of cancer, if present (based on WHO classification [16]); Gleason score (GS) with primary, secondary and tertiary, if appropriate, grades (according to 2005 ISUP Consensus Conference [17]); pathological stage [18]; evaluation of tumour extension, local invasion into periprostatic tissue or seminal vesicles, perineural spread, venous and/or lymphatic vessel invasion and surgical margin status [19]. PCa was present in 329 surgical specimens, 105 were classified as BPH without any malignant structures. Table 2 summarizes GS and staging information for the PCa group. Fasting peripheral venous blood samples were drawn between 6 and 8 a.m. on the day of surgery and centrifuged at 2000 rpm for 20 minutes. The aliquots were kept frozen at -80°C until analysis.

**Table 2:** Gleason score and tumour stage in prostate cancer cases

Tumour grading	
Median Gleason score	6

2-6	169 (51.4%)

7	124 (37.7%)

8-10	36 (10.9%)

**Tumour pathological stage**	

≤pT2a	18 (5.5%)

pT2b	5 (1.5%)

pT2c	134 (40.7%)

pT3a	98 (29.8%)

≥pT3b	63 (19.1%)

pTX	11 (3.4%)

### Laboratory analyses

The presence of antibodies to Chlamydia trachomatis (C. trachomatis), herpes simplex virus (HSV) 1 and 2, human cytomegalovirus (CMV), Neisseria gonorrhoeae (N. gonorrhoeae), Treponema pallidum (T. pallidum), Mycoplasma hominis (M. hominis) and Ureaplasma urealyticum (U. urealyticum) were detected by means of enzyme-linked immunoanalysis (ELISA), complement fixation test (CFT) or indirect immunofluorescence (IF) using commercially available, CE marked kits according to the manufacturers' recommendations. Table 3 gives a detailed description of the kits. Specific antibodies to HPV were detected by means of an in-house method using virus-like particles (prepared in a recombinant baculovirus/insect cells system) as antigens [20]. Laboratory personnel were blinded to case/control status of the samples.

**Table 3:** Commercially available kits used for the detection of antibodies

Pathogen	Method	Kit	Manufacturer
Cytomegalovirus	ELISA	ETI-CYTOK-G	DiaSorin S.p.A., Saluggia, Italy

Herpes simplex virus type 1	ELISA	Captia™HSV 1 IgG	Trinity Biotech plc, Bray, Ireland

Herpes simplex virus type 2	ELISA	HerpeSelect(R) 2 ELISA IgG	FOCUS Diagnostics, Cypress, CA, USA

Chlamydia trachomatis	ELISA	Chlamydia Trachomatis IgG - ELISA	NovaTec Immunodiagnostica GmbH, Dietzenbach, Germany

Mycoplasma hominis	Indirect immunofluorescence test	Anti-Mycoplasma Hominis IIFT (IgG)	EuroImmun, Lübeck, Germany

Ureaplasma urealyticum	Indirect immunofluorescence test	Anti-Ureaplasma Urealyticum IIFT (IgG)	EuroImmun, Lübeck, Germany

Neisseria gonorrhoeae	Complement fixation test	Complement fixation test Neisseria gonorrhoeae	Serion Immunodiagnostica GmbH, Würzburg, Germany

Treponema pallidum	ELISA	Syphilis EIA II (TA]	Newmarket Laboratories Ltd, Kentford, UK

### Preparation of virus-like particles (VLPs)

Insect cells Sf9 were grown in suspension cultures in TNM-FH insect medium (Sigma). About 4 × 10^9 ^of cells were infected by recombinant baculovirus at MOI 10 PFU per cell. Various recombinant baculoviruses carried the genetic information for L1 capsid protein of specific HPV types. Seventy-two hours after infection, the cells were harvested, washed with phosphate-buffered saline (PBS) and stored at -20°C. Cell pellets were resuspended in 40 ml of extraction buffer (10 mmol/l MgCL_2_, 50 mmol/l CaCl_2_, 150 mmol/l NaCl, 0.01% Triton X-100, 20 mmol/l HEPES, pH 7.4) and sonicated 3x30 minutes on ice, the suspension was pelleted, the pellet was resuspended in 40 ml of extraction buffer and sonication was repeated. Cesium chloride was added to combined supernatants to the final concentration of 30% and ultra-centrifuged for 22 hours at 45,000 rpm at 18°C. The upper band was then separated by ultra centrifugation on CsCl step gradient (36%-30.5%-16%) for 4 hours at 35,000 rev/min. at 18°C. The band was removed and subjected to SDS PAGE to determine protein concentration and Western blotting, and ELISA with corresponding VLP-specific monoclonal antibodies to prove the presence of the respective antigen.

### Detection of HPV-specific antibodies

The presence of antibodies to the antigens derived from HPV-specific proteins was tested using the enzyme-linked immunosorbent assay (ELISA). VLPs mimicking HPV types 6, 11, 16, 18, 31 and 33 were used as antigens. Briefly, wells of microtiter plates (Polysorp NUNC immunoplate, Thermo Fisher Scientific, Denmark) were coated with 50 μl of purified VLP (2 μg/ml) in PBS at 37°C for 2 hours and at +4°C overnight. All subsequent incubations were performed at +37°C for 1 hour. Unbound antigen was removed, nonspecific binding sites were blocked by incubation with 1% bovine-serum albumin (BSA) in PBS, and the wells were incubated in duplicate with 100 μl of human sera diluted 1:25 in washing buffer (PBS, 0.21 mol/l NaCl, 0.1% Triton X-100) with 1% of BSA. Following incubation, the antibodies bound were detected with donkey anti-human IgG (H and L chain), covalently linked to horseradish peroxidase (Jackson ImmunoResearch Laboratories, Inc, West Grove, PA, USA), in the same buffer (1:7,500) and the reaction was visualized by adding 100 μl of a substrate buffer (50 mmol/l phosphate-citrate buffer, pH 5.0) containing 0.04% o-phenylenediamine and 0.006% hydrogen peroxide. The color reaction was stopped by 100 μl of 2 mol/l H_2_SO_4 _and optical densities at 492 and 630 nm were read with an Infinite 200 microplate reader (TECAN, Austria). Background reactivity was determined in wells without antigen. Their absorbances were subtracted from the corresponding values obtained in the presence of antigen. Control sera known to be positive or negative were tested on each plate. The cut-off (CO) level, above which the optical density values were considered positive, was represented by a mean absorbance plus 2 standard deviations (SD) after eliminating the outliers - samples with absorbances higher than the calculated cut-off value - and the calculation was repeated until the absorbances of all remaining samples were lower. The means and SDs were calculated for each antigen/plate separately. All ELISA results are represented as a ratio between the absorbance obtained with the tested sample and the appropriate cut-off value (OD index), which expresses the strength of antibody response (antibody levels). To confirm the results, all samples within 10% above the CO value as well as about one quarter of all serum samples were retested. Samples with OD index values ≤ 1.0 were considered not reactive.

### Statistical analyses

Descriptive statistics was employed to assess demographic and clinical variables in all subjects. Local stage and tumour aggressiveness by GS were calculated in PCa patients.

We established seropositivity rates for each individual pathogen in cases and in controls. Percentages of seropositive individuals were compared using Fisher's exact test.

The mean serum antibody levels represented by OD indexes were calculated and compared between the two groups for HPV, HSV, CMV and C. trachomatis where ELISA allows for quantitative assessment using Wilcoxon's (nonparametric) test.

We were looking for a possible association between seropositivity and PSA level, seropositivity and tumour GS, and seropositivity and localized vs. locally advanced disease using appropriate logistic regression models. Throughout the paper, 5% significance level is meant whenever we mention statistical significance. All statistical analyses were computed using statistical software "R" http://www.r-project.org/.

This paper's contents were developed with regard to current recommendations on reporting of observational studies [21].

## Results

In a total of 434 study subjects, there were 329 PCa patients (mean age 64 years) and 105 controls with BPH (mean age 72 years). PCa had been present in 11 patients out of 116 who underwent SP. These incidental carcinomas were included in the PCa group for case vs. control comparisons but were excluded from analyses where exact local staging was required (see Figure 1). The mean PSA levels were 9.0 and 7.5 ng/mL among subjects and controls, respectively (Table 1). Among PCa patients, 47.7% were diagnosed with localized disease and 52.3% had locally advanced cancer. One half of the tumours (51.4%) were low-grade lesions GS 2-6, while one tenth (10.9%) were high-grade lesions GS 8-10 (Table 2).

**Figure 2:**
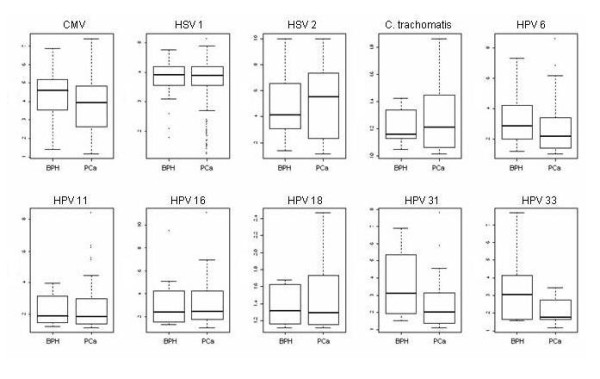
**Mean serum antibody levels**. Boxplots comparing serum antibody level medians (thick horizontal lines) of cases and controls. Upper and lower horizontal box lines represent the 25^th ^and 75^th ^percentiles. Outliers are marked as dots. CMV human cytomegalovirus, HSV herpes simplex virus, HPV human papillomavirus, BPH benign prostate hyperplasia, PCa prostate cancer.

The seropositivity rates and mean serum antibody levels are summarized in Table 4. U. urealyticum antibodies were significantly more common in PCa cases than in controls (OR 2.06; 95% CI 1.08-4.28). Conversely, the seropositivity rates for HPV 18 and C. trachomatis were significantly higher in BPH patients than in the PCa group using Fisher's exact test (OR 0.23; 95% CI 0.09-0.61 and OR 0.45; 95% CI 0.21-0.99, respectively). The mean serum antibody levels for CMV were higher in the BPH than in the PCa group using Wilcoxon's test (4.407 vs. 3.839; p = 0.0004; see Table 4). For other pathogens, the differences in the seroprevalence and mean serum antibody levels were not statistically significant (Figure 2).

**Table 4:** Antibody seropositivity rates and mean serum antibody levels

	Seroprevalence rate				Mean serum levels OD/CO		
	**PCa**	**BPH**	**OR**	**95% CI**	**PCa**	**BPH**	**p**

*HPV 6*	77 (24.1%)	21 (20.8%)	1.21	0.71-2.12	2.608	3.162	0.0964

*HPV 11*	40 (12.5%)	15 (14.9%)	0.82	0.44-1.60	2.554	2.259	0.9698

*HPV 16*	16 (5.0%)	10 (9.9%)	0.48	0.21-1.13	3.470	3.251	0.8743

*HPV 18*	8 (2.5%)	10 (9.9%)	0.23	0.09-0.61	1.496	1.365	0.9290

*HPV 31*	19 (5.9%)	8 (7.9%)	0.73	0.32-1.83	2.616	3.640	0.1053

*HPV 33*	7 (2.2%)	5 (5.0%)	0.43	0.13-1.48	2.156	3.620	0.3434

*CMV*	264 (80.2%)	91 (86.7%)	0.62	0.32-1.14	3.839	4.407	0.0004

*HSV 1*	313 (95.1%)	99 (94.3%)	1.19	0.42-2.97	3.760	3.832	0.9649

*HSV 2*	42 (12.8%)	14 (13.3%)	0.95	0.51-1.88	5.362	4.859	0.8056

*Chlamydia trachomatis*	18 (5.5%)	12 (11.4%)	0.45	0.21-0.99	1.281	1.210	0.6560

*Mycoplasma hominis*	60 (18.2%)	15 (14.3%)	1.34	0.74-2.55	NA	NA	-

*Ureaplasma urealyticum*	64 (19.5%)	11 (10.5%)	2.06	1.08-4.28	NA	NA	-

*Neisseria gonorrhoeae*	20 (6.1%)	6 (5.7%)	1.07	0.44-2.99	NA	NA	-

*Treponema pallidum*	1 (0.3%)	0 (0%)	NA	NA	NA	NA	-

Antibody seropositivity rates for different genitourinary pathogens in cases and controls. Only reactive serum samples (OD/CO > 1.0) were considered for the calculation of mean serum antibody levels. PCa prostate cancer, BPH benign prostate hyperplasia, OR odds ratio, CI confidence interval, OD/CO optical density/cut-off, HPV human papillomavirus, CMV human cytomegalovirus, HSV herpes simplex virus, NA not applicable

We then analyzed the PCa cases alone with regard to serostatus and disease characteristics. Patients seropositive for HPV 6 were more likely to have higher GS than men without antibodies to HPV 6 (mean GS 6.45 vs. 6.75; p = 0.0305); other differences in GS were not demonstrated. No statistically significant difference was noted in the mean PSA level between the seropositive and seronegative (for each pathogen) individuals. The mean serum antibody levels did not differ significantly between the patients with localised disease (≤pT2c) and locally advanced PCa (≥pT3a). These observations did not change when age was taken as an additional variable and linear/logistic regression models were employed (data not shown).

## Discussion

Clinically insignificant foci of PCa are harboured by prostates of 80% of men over 80 years old [22]. The only way to examine the prostate in its entirety and to divide the subjects into the case and control groups is the use of open surgery derived specimens. This approach has not been used so far. No needle biopsy or TURP which can only deliver a small portion of prostatic tissue allows the clinician to rule out the possibility of cancer being present in the rest of the prostate gland. Approximately 1/10 of allegedly benign prostates scheduled by a surgeon for SP turned out as cancerous in our study, which is in line with the 4-20% incidental prostate cancer rate cited in the literature [23]. Most of the previous studies identified cases in cancer registries while controls were defined either by absence of PCa diagnosis [7,24-27] or as "cancer free" at the time of analysis [6,8,28]. This approach can be misleading, because it is the nature of the disease to evolve as subclinical lesions for years or even decades and inclusion of these patients into the control group may have biased the results towards the null. Only few authors assessed the controls more deeply, i.e. by histopathologic examination of prostate tissue [3-5,29,30] obtained by open surgery, TURP or prostate biopsy. Inherently, a needle biopsy or TURP can only confirm but by no means exclude the diagnosis of cancer. Some researchers gathered details of tumour characteristics such as GS and staging by TNM classification [5,8,26,28-32], but in most cases they do not report these data.

The exclusive use of radical prostatectomy-derived specimens allowed for the assessment of seropositivity with regard to exact local staging of the disease. This is the first study to our knowledge to look at the issue so closely. Had there been an association between local disease stage and serostatus, it would have been demonstrated. However, no statistically significant differences were noted. Similarly, no differences were found in the mean PSA levels and mean GS between the seropositive and seronegative PCa patients (with the exception of HPV 6, as mentioned above and discussed later on in this section). The picture remained the same when age was taken as an additional variable.

Since the 1950s when Ravich and Ravich [33] hypothesized that PCa could be caused by an infectious agent, research studies have been conducted in order to confirm or rule out this possibility. Taylor et al. reviewed the literature related to sexually transmitted diseases (STDs) and PCa from 1966 to 2004 identifying 6022 unique PCa cases and 7320 controls and concluded that having had any STD was associated with an elevated PCa risk: odds ratio (OR) of 1.48, 95% confidence interval (CI) 1.26-1.73 [34]. A large recent prospective study by Huang et al., however, did not find an association of PCa with a specific STD and a borderline association with any vs. none [26]. Whereas C. trachomatis, HPV 16 and 18, HSV 2, CMV and human herpesvirus 8 were ascertained by serology in this study, a history of syphilis and gonorrhoea was only self-reported.

HPV is a sexually transmitted pathogen which has been the most extensively investigated one because of its association with cervical and a part of other anogenital neoplasms [32]. We have identified 12 epidemiological studies related to HPV and PCa, a vast majority of them being case-control studies putting together over 4,700 PCa cases and 7,300 controls. No more than two of them [24,29] found an association between PCa and HPV 18 and 33, respectively, whereas the others did not (see Table 5 for details). In our study population, HPV 18 antibodies were more common in men with BPH than in PCa patients. Such an inverse association has not been reported so far.

**Table 5:** Overview of previously published case-control serologic studies of genitourinary pathogens with regard to prostate cancer risk

Author	Year	Pathogen studied	No. of subjects with PCa	% sero-positive	No. of controls without PCa	% sero-positive	RR/OR	95% CI	Evidence of association	Method
Sutcliffe [30]	2010	HPV 16	616	14.5	616	13.7	OR 1.07	0.77-1.48	no	ELISA
			
		HPV 18	616	3.3	616	3.7	OR 0.87	0.47-1.63	no	
			
		HPV 31	616	12.3	616	10.8	OR 1.15	0.8-1.64	no	

Dennis [27]	2009	HPV 16/18	267	18.7	267	16.9	OR 1.13	0.73-1.75	no	ELISA

Huang [26]	2008	HPV 16	765	10.1	915	10.6	OR 0.9	0.7-1.3	no	ELISA
			
		HPV 18	765	9.4	915	8.1	OR 1.2	0.8-1.7	no	

Sitas [9]	2007	HPV 16	205	68.0	673	58.0	OR 1.33	0.86-2.07	no	ELISA

Sutcliffe [28]	2007	HPV 16	691	7.5	691	8.8	OR 0.83	0.57-1.23	no	ELISA
			
		HPV 18	691	6.1	691	5.8	OR 1.04	0.66-1.64	no	
			
		HPV 33	691	7.2	691	6.4	OR 1.14	0.76-1.72	no	

Korodi [8]	2005	HPV 16	799	6.0	2596	6.0	OR 0.9	0.64-1.26	no	ELISA
			
		HPV 18	799	3.0	2595	4.0	OR 0.79	0.49-1.26	no	
			
		HPV 33	800	9.0	2596	7.0	OR 0.99	0.72-1.38	no	

Adami [29]	2003	HPV 16	238	13.0	210	15.0	OR 0.7	0.4-1.3	no	ELISA
			
		HPV 18	238	12.0	210	12.0	OR 0.9	0.5-1.9	no	
			
		HPV 33	238	29.0	210	23.0	OR 1.6	1.0-2.7	yes	

Rosenblatt [32]	2003	HPV 16	642	9.2	570	8.8	OR 1.06	0.71-1.57	no	ELISA
			
		HPV 18	642	3.4	570	2.5	OR 1.36	0.69-2.69	no	

Hayes [31]	2000	HPV 16	276	6.9	295	5.1	OR 1.4	0.7-2.8	no	ELISA

Hisada [6]	2000	HPV 16	48	42.0	63	30.0	OR 2.7	0.9-7.9	no	ELISA

Dillner [23]	1998	HPV 16	165	4.0	290	2.0	RR 2.4	0.75-7.58	no	ELISA
			
		HPV 18	165	10.0	290	4.0	RR 2.6	1.17-5.75	yes	
			
		HPV 33	164	4.0	289	6.0	RR 0.7	0.26-1.66	no	

Strickler [5]	1998	HPV 16	63	1.6	144	4.9	NS	NS	no	ELISA

Dennis [26]	2009	Ch. trachomatis	267	14.6	267	11.6	OR 1.35	0.79-2.31	no	Microimmuno-fluorescence

Huang [25]	2008	Ch. trachomatis	765	11.2	915	9.7	OR 1.2	0.9-1.6	no	ELISA

Sutcliffe [27]	2007	Ch. trachomatis	691	4.0	691	3.5	OR 1.13	0.65-1.96	no	ELISA

Antilla [7]	2005	Ch. trachomatis	738	7.5	2271	10.5	OR 0.69	0.51-0.94	yes*	Microimmuno-fluorescence

Dillner [24]	1998	Ch. trachomatis	165	10.9	290	10.7	RR 1.04	0.54-2.00	no	Microimmuno-fluorescence

Hayes [31]	2000	T. pallidum	271	10.7	286	6.3	OR 1.8	1.0-3.5	yes	Microhemagglut-ination assay (MHA-TP)

Dennis [27]	2009	HSV 2	267	28.5	267	20.6	OR 1.6	1.05-2.44	yes	ELISA

Huang [26]	2008	HSV 2	765	9.2	915	9.7	OR 0.9	0.7-1.3	no	enzymatic immunodot assay

Korodi [25]	2005	HSV 2	163	7.2	288	7.5	OR 0.93	0.44-1.96	no	ELISA

Baker [4]	1981	HSV 2	50	68.0	159	51.0	NS	NS	yes	indirect hemagglutination inhibition test

Herbert [3]	1976	HSV 2	28	71.4	29	65.5	NS	NS	no	microcomplement fixation test

Huang [26]	2008	CMV	765	70.3	915	68.4	OR 1.1	0.9-1.3	no	ELISA

OR odds ratio, RR relative risk, CI confidence interval, HPV human papillomavirus, HSV herpes simplex virus, CMV human cytomegalovirus, ELISA Enzyme-linked Immunosorbent Assay, NS not specified, * inverse association

The seropositivity rates reported in our study were 5.0% and 2.5% in PCa patients for HPV 16 and 18, respectively, and 9.9% for both antigens in the BPH group (Table 4). This coincides well with the estimated 10.2% HPV 16 seroprevalence in the US male population aged 50-59 years [35].

HPV 6 is associated with benign skin lesions of the genitourinary tract. This genotype was demonstrated in up to 90% of genital warts [36]. To our knowledge, no previous study investigated the association of HPV 6/11 and PCa nor suggested the virus' potential to induce a more malignant phenotype of PCa cells. However, these two serotypes have been associated with malignant transformation of recurrent respiratory papillomatosis [37]. Our finding of a higher mean GS, i.e. increased aggressiveness of the prostate tumour in the presence of HPV 6 antibodies, would require further validation.

Infection with certain herpesviruses has been associated with several human cancers. A link has been found between HSV 1 and oral cancer, while HSV 2 seems to be a cofactor to HPV in the etiology of invasive cervical cancer [38]. Sexual transmission of HSV 1 and 2 leading to infection of the genitourinary tract is a commonplace. HSV 2 is seldom completely cleared from the body. Antibody levels may fluctuate over time, especially after clinical relapses, but the exact dynamics is not known [27]. In the U.S., 51.0% of persons over 12 years were seropositive for HSV 1, 5.3% for HSV 2 and in 16.6%, antibodies specific to both virus types were present [39]. The HSV 2 prevalence is highest in some African countries, reaching 80% in persons ≥35 years [40].

Sexual contact is a major route of CMV transmission in adults. Increased CMV rates are associated with increased history of sexually transmitted diseases. Recent data indicate that CMV has multiple oncogenic properties: it promotes mutagenesis, angiogenesis and cell invasion [41]. The CMV seroprevalence increases with age, reaching approximately 91% among persons older than 80 years [42]. Little is known about the relationship between CMV and PCa risk. The only relevant study we have identified did not show evidence of an association of CMV seropositivity and PCa incidence [26]. Our data suggest that PC cases have lower antibody levels against CMV than BPH patients.

C. trachomatis is one of the most common bacterial STDs worldwide. It causes urethritis, epididymitis and prostatitis in males. In up to 50% of infected men and 70-80% of infected women, the infection is clinically inapparent [43]. In four recent epidemiological studies of several hundred cases and controls each [24,26-28], no association between chlamydial antibodies and PCa was found. One large study [7], however, shows a significant inverse association (OR 0.69, 95% CI 0.51-0.94) between chlamydial antibodies and PCa and so do our data. The seroprevalence we have established is concordant with the data reported previously [44].

The increased antibody prevalence and higher serum antibody levels we are reporting do not suggest the role of infection in PCa pathogenesis. They do indirectly support a concept of BPH being product of immune inflammatory processes, an idea based on a growing amount of evidence [45]. The trigger point for an increased and possibly self-repeating, pathological immune response would be infection of the genitourinary tract by these pathogens [46].

A study of Takeyama suggested that M. hominis can trigger inflammation in the prostate by inducing interleukin-8 secretion [47]. Chronic infection by M. genitalium and M. hyorhinis of human prostate cells resulted in alteration of karyotypes (notably increased polysomy) and malignant transformation in vitro. Inoculated in nude mice, these cell lines initiated tumour growth [48]. We have not identified any study concerned with the clinical association between PCa and Mycoplasma and/or Ureaplasma. To our knowledge, we are the first to report an increased seroprevalence of U. urealyticum antibodies in PCa patients compared to men with BPH.

Gonorrhoea and syphilis are historically most notorious STD's. In a large prospective cohort study of Sutcliffe et al., gonorrhoea and syphilis were self-reported in 3% and 0.2%, respectively, and no relationship with PCa was observed [49]. Self-report of gonorrhoea and/or syphilis was associated with PCa (OR 1.6, 95% CI 1.2-2.1) in a study of Hayes et al. Of note, however, serologic evidence of T. pallidum infection was found in 9.9% vs. 2% reported (in the black population) and 3.2% vs. 0.1% reported (in whites) [31]. This fact illustrates how unreliable questionnaire and interview-based studies can be whether for recall bias, unwillingness to report a socially embarrassing disease such as STD or just by ignorance, i.e. failing to classify a condition as STD.

Our seroprevalence data for T. pallidum and N. gonorrhoeae are comparable for PCa and BPH patients. However, the statistical power is low due to the small number of patients to draw any reasonable conclusions.

Every research paper has its limitations and so does ours. Because blood samples were drawn after the diagnosis of PCa, we cannot state whether contact with the pathogen preceded or followed the evolution of cancer. Taking BPH patients as controls brings the risk of not coming to a meaningful conclusion if both conditions had the same (shared) etiology. On the other hand, virtually no healthy prostates exist in the age group our patients belong to.

## Conclusions

This is the first study to our knowledge to demonstrate a higher U. urealyticum seroprevalence rate in PCa patients when compared to BPH controls. HPV 18 and C. trachomatis seropositivity was more common and the mean CMV antibody levels were higher in the control (BPH) group than among PCa cases. Unlike other research groups, we used open surgery-derived specimens exclusively for all analyses. This allowed us 1) to minimise the likelihood of misclassifying a tissue sample as benign if cancer was present and 2) to look for a correlation of PCa and infection in subgroups of patients depending on local disease stage. This novel type of analysis did not demonstrate any differences.

Disregarding inconsistencies of results of the previous studies, this one adds to the growing body of evidence that the presence or higher levels of serum antibodies to most of the genitourinary pathogens studied are not associated with an elevated PCa risk and/or a more malignant tumour behavior. Antibody seropositivity to these infectious agents does not emerge as a risk factor in screening or treatment decision making. Tissue analyses focused on viral or bacterial DNA presence could be a track to run to cast light onto possible association between PCa and infectious agents.

## Competing interests

The authors declare that they have no competing interests.

## Authors' contributions

JHr participated in the study design, recruited the patients, reviewed the literature and drafted the manuscript. JHe and MU conceived the study design, recruited the patients and reviewed the whole manuscript. MU performed most of the surgeries. EH and RT carried out the immunoassays and wrote the appropriate part of the Methods section. VE carried out the histopathological examinations. MB performed the statistical analyses and covered that part in the text. All authors read and approved the final version of the manuscript.

## Authors' information

JHr is a PhD student of the 3^rd ^Faculty of Medicine, Charles University, Prague; this work represents a part of his thesis. JHe is a research fellow at the same institution. EH is the head of the Laboratory for Prevention of Viral Infections, Institute of Hematology and Blood Transfusion. MU is the head of the Department of Urology of the 3^rd ^Faculty of Medicine, Charles University, Prague.

## Pre-publication history

The pre-publication history for this paper can be accessed here:

http://www.biomedcentral.com/1471-2407/11/53/prepub
